# Clinical observation of transurethral reconstruction of urethral meatus and navicularis fossa short strictures with annular inlay oral mucosa graft urethroplasty

**DOI:** 10.3389/frph.2025.1555597

**Published:** 2025-08-05

**Authors:** Zhenyu Li, Zhiguang Zhao, Geng Zhang, He Wang, Bo Zhang, Wei Zhang

**Affiliations:** Department of Urology, Tangdu Hospital, Air Force Military Medical University, Xi’an, China

**Keywords:** urethral stricture, navicular fossa, oral mucosa, urethroplasty, annular inlay

## Abstract

**Objective:**

This study aims to explore the clinical efficacy and safety of a urethroplasty technique for fossa navicularis (FN) strictures using a transurethral annular inlay oral mucosa graft.

**Methods:**

A retrospective analysis was conducted on clinical data from patients with urethral meatus and navicular fossa stricture who underwent transurethral reconstruction using annular inlay oral mucosa graft urethroplasty in Tangdu Hospital from July 2021 to October 2024. Operation success was defined as the ability to pass the F24 urethral probe, and the secondary outcome is the urinary flow rate and patient satisfaction at 1, 3, 6, and 12 months.

**Results:**

All 12 patients successfully completed the surgery. The average age was 56.8 ± 6.8 years, and the average length of urethral stricture was 1.6 ± 0.2 cm. Three patients had a history of transurethral endoscopic surgery, eight had penile lichen sclerosus (LS), and one had no obvious causes. Over a median follow-up of 19 months, the average maximum urinary flow rate was 21.2 ± 3.2 ml/s at 3 months, and the average maximum urinary flow rate was 19.5 ± 4.2 ml/s at 12 months. One patient experienced urine pain with a thin stream and spraying urination after 3 months. Physical examination of the urethral meatus opening scar was found, and the symptoms disappeared after urethral incision. The follow-up survey of sexual life after 1 year showed that seven patients (58.3%) had successful life within 1 year compared with two patients (16.7%) before surgery. There were no cases of erectile dysfunction and poor wound healing. The patients were either very satisfied (75%) or satisfied (25%) with the operation. All patients would recommend urethroplasty to others.

**Conclusion:**

Transurethral annular inlay oral mucosa urethroplasty for urethral meatus and navicular fossa stricture reconstruction is a safe, feasible, and effective surgical method, cosmetic effect on the penile head. This technique has advantages in improving sexual function and better penile head cosmesis.

## 1 Introduction

Urethral meatus and navicularis fossa strictures represent a specific form of urethral stricture, constituting approximately 18% of all urethral strictures. The etiological factors encompass lichen sclerosus (LS), traumatic injury, iatrogenic damage, and urethral stricture secondary to hypospadias repair, among others. Despite the relatively short length of this type of urethral stricture, it exhibits a high restenosis rate. Additionally, patients have more expectations for the recovery of appearance and sexual function, as well as for minimizing complications (1–3). In the past, simple urethral strictures could be managed through urethrotomy. But for patients with extensive stenotic segments, previous approaches included ventral transverse or annular incisions of the coronal sulcus, along with oral mucosa grafting or urethral reconstruction using a pedicled flap based on individual conditions. These methods often resulted in unsatisfactory cosmetic outcomes and required penile corpora spongiosum longitudinal incision to reconstruct the glans. Patients with LS stenosis are at high risk for recurrent stenosis, and local skin reconstruction is generally not advisable (4). In recent years, transurethral oral mucosa transplantation has gained recognition as a viable treatment for urethral meatus and scaphoid fossa stenosis. This article details the surgical technique of complete replacement urethroplasty with annular inlay oral mucosa graft. In this study, we retrospectively reviewed the clinical data of 12 male patients with urethral meatus and navicularis fossa strictures who were admitted to the Department of Urology at Tangdu Hospital, Air Force Military Medical University, from July 2021 to October 2024. This study aims to evaluate the efficacy and safety of this surgical approach.

## 2 Material and methods

### 2.1 Material

A retrospective analysis of the patient database identified male individuals with urethral meatus and navicularis fossa strictures admitted to the Department of Urology at Tangdu Hospital, from July 2021 to July 2024. All urethroplasties were performed by a single surgeon. Patients with strictures that extended proximal to the fossa navicularis, those with synchronous urethral strictures at a separate location, and those with hypospadias were excluded. The study was approved by our institutional review board. Preoperative evaluation included detailed medical history documentation, physical examination, and imaging assessment with retrograde urethrography. Patient-reported outcomes were systematically assessed using the validated American Urological Association (AUA) Symptom Score questionnaire, with higher scores indicating more severe voiding dysfunction. Patients were followed up at the outpatient clinic at 1, 3, 6, and 12 months postoperatively. Follow-up assessments included uroflowmetry, documentation of successful sexual intercourse episodes, and satisfaction scores. Objective voiding parameters were measured through standardized uroflowmetry testing [maximum flow rate (Qmax), voided volume] and postvoid residual (PVR) urine quantification via transabdominal ultrasound.

### 2.2 Surgical procedure

**Figure 1:**
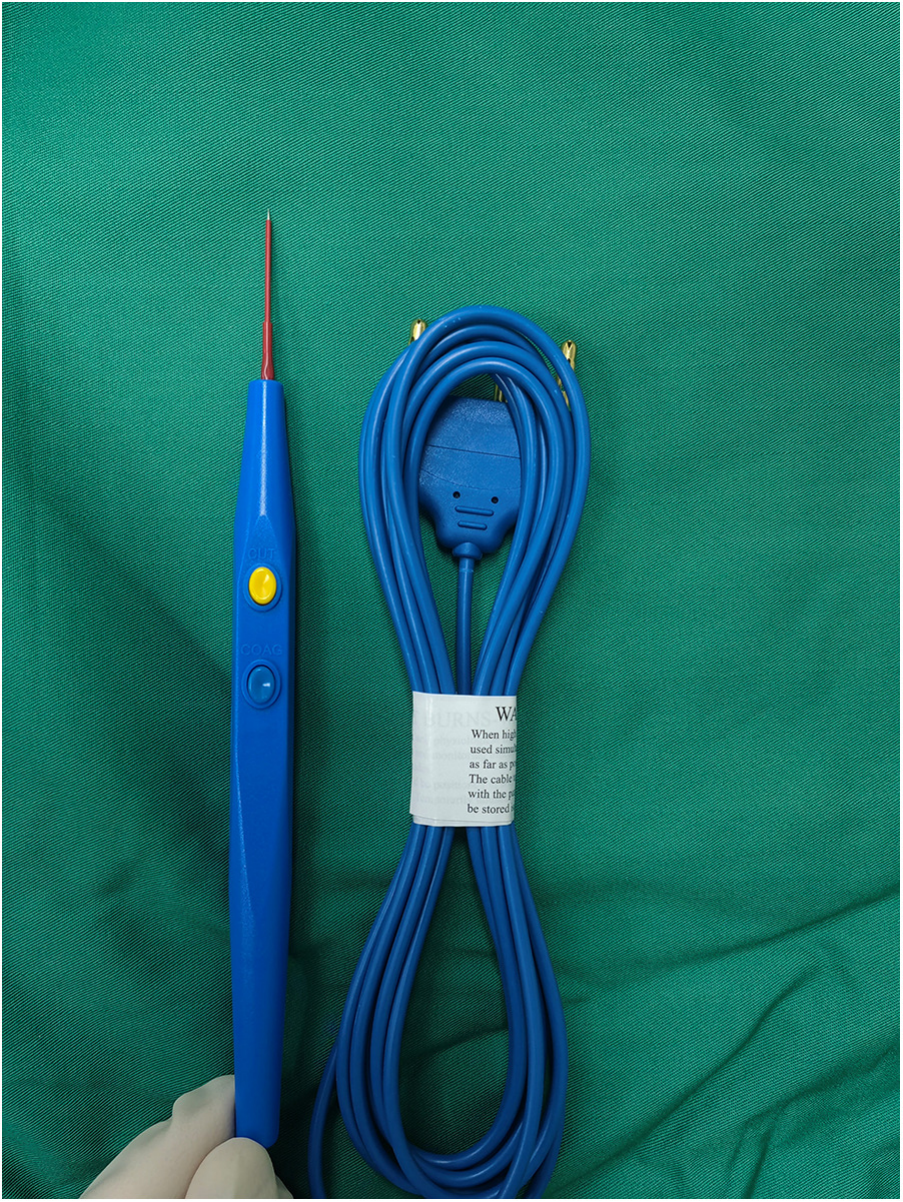
High-frequency microneedle electrode.

All patients underwent a 3-day preoperative preparation, which included gargling with compound chlorhexidine solution and metronidazole sodium chloride. Additionally, the perineal area was thoroughly cleaned and prepared. Under general anesthesia with nasal intubation, the patients were in the lithotomy position. The urethra was irrigated with a 1:2 dilution of an iodophor solution, followed by disinfection and draping. The high-frequency microneedle electrode (Figure 1) was prepared; it demonstrated superior performance to ophthalmic scissors for fine urethral incisions.

**Figure 2:**
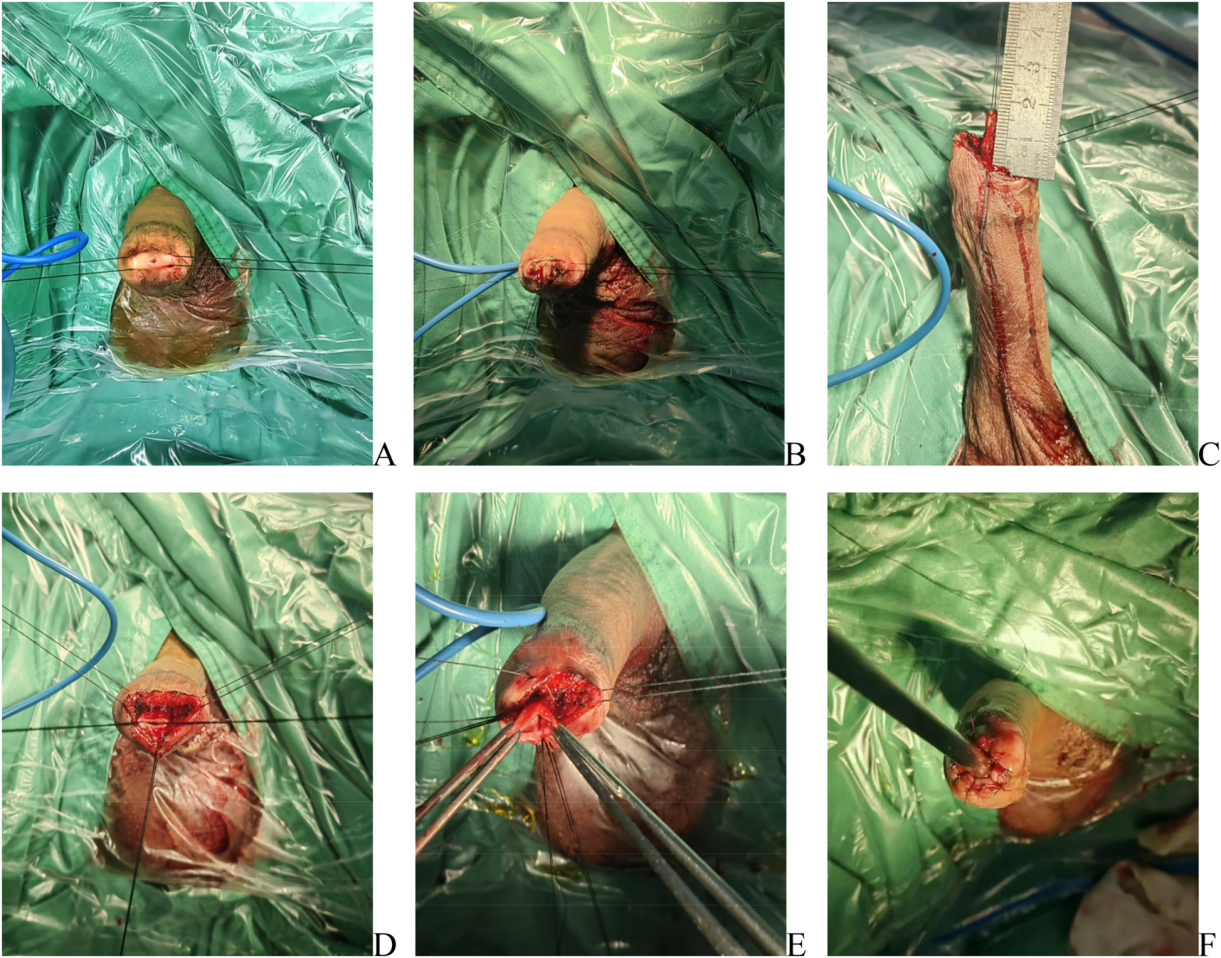
Surgical steps of transurethral annular inlay oral mucosa graft urethroplasty for navicular fossa strictures. **(A)** Traction established with two 3-0 silk sutures through the glans. **(B)** Two additional 3-0 silk sutures were placed at the 3- and 9-o’clock positions of the urethral meatus to provide traction; a circumferential incision was made lateral to the corpus spongiosum. **(C)** Stricture length measured intraoperatively using a calibrated scale. **(D)** Excision of the stenotic segment; three 3-0 silk traction sutures were placed in the normal urethral mucosa to facilitate graft anastomosis. **(E)** Oral mucosa graft secured to the proximal apex of the urethral defect with six interrupted 5-0 absorbable sutures. **(F)** Postoperative calibration confirming patency with an F24 urethral steel sound.

Two 3-0 silk traction sutures were placed through the glans and used to stretch the penis (Figure 2A). Two additional 3-0 silk sutures were then placed at the 3- and 9-o’clock positions of the urethral meatus to provide traction. Subsequently, using the high-frequency needle electrode, a circumferential incision was made lateral to the corpus spongiosum (Figure 2B). The urethra was sounded with a urethral sound, traversing the stricture until normal urethral mucosa distal to the stricture segment was reached. Subsequently, the length of the urethral stricture was measured using a ruler (Figure 2C). The urethral stricture segment was excised using tenotomy scissors. The normal urethral lumen at the distal end was exposed, ensuring easy passage of the F24 urethral steel. Three 3-0 silk traction sutures were placed in the normal urethral mucosa to facilitate subsequent anastomosis with the oral mucosa graft (Figure 2D). Based on the measured dimensions, an appropriately sized oral mucosa graft was harvested. The submucosal fat layer was removed, and the mucosa was punched out. Six 5-0 absorbable sutures were placed through the normal urethral mucosa at the 0-, 2-, 4-, 6-, 8-, and 10-o'clock positions. Subsequently, the opposite ends of these sutures were threaded sequentially onto a small round needle and passed through the distal end of the oral mucosa graft. The sutures were then progressively tightened to approximate the oral mucosa graft into the urethral lumen, covering the defect. Internal fixation was achieved by tying the sutures within the mucosa (Figure 2E). The edges of the oral mucosa graft and the distal urethral mucosa were discontinuously sutured with 5-0 absorbable sutures, while the central portion of the graft overlying the urethral defect was similarly secured. This ensured stable fixation of the graft to the urethral bed. The urethra accommodated an F24 urethral steel (Figure 2F). Intraoperatively, an F16 three-lumen silicone catheter was placed, and a compression dressing was applied to the glans penis to reduce edema.

### 2.3 Postoperative status and follow-up care

**Figure 3:**
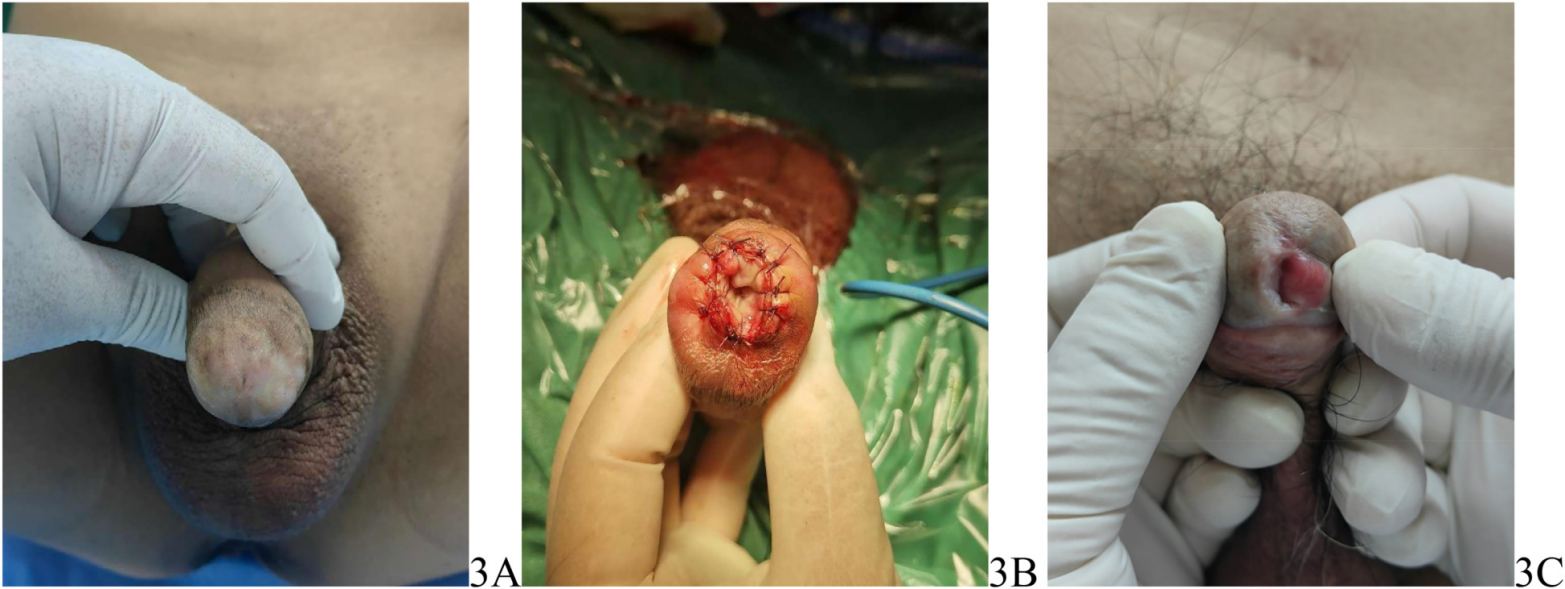
**(A)** Preoperative appearance of the penis, **(B)** postoperative appearance of the penis, **(C)** the appearance of the glans 3 months postsurgery.

After surgery, the patient's urethra was spacious (Figures 3A,B). The elastic bandage was removed on postoperative day 3. Daily observations were conducted to monitor the healing of the oral mucosa, and the urethral meatus was disinfected daily using iodophor swabs. The catheter was removed two weeks after surgery. Follow-up visits were scheduled at 1 month and 3 months postoperatively to assess the patient's urinary patency and check for any recurrence of strictures. During these visits, the healing of the mucosa and the color and appearance of the penile head were also evaluated. Three months after surgery, urine flow rate was measured, with an average maximum urine flow rate of 21.2 ± 3.2 ml/s. At the 3-month follow-up, a patient experienced dysuria, a narrowed urinary stream, and spraying urination. Physical examination revealed recurrent LS at the reconstructed urethral meatus, accompanied by scar formation. The patient underwent a urethral meatotomy and was then prescribed a standardized postoperative regimen: daily application of 0.05% clobetasol propionate ointment to the glans penis, along with weekly self-dilation using clobetasol-coated urethral sounds (16–18 Fr). The dilation interval was gradually increased to a monthly maintenance schedule after 3 months of treatment, leading to complete resolution of symptoms. This case underscores the significance of postoperative prophylactic management with topical clobetasol in patients with LS-associated meatal strictures, indicating that regular application to both the glans and urethral mucosa may reduce the risk of recurrence by targeting the underlying inflammatory process. All other patients reported satisfactory urination and were content with the appearance of the postoperative penile head, noting that the external urethral meatus appeared slit-like. The patients were satisfied with the appearance of the glans (Figure 3C).

## 3 Result

All 12 patients underwent successful surgical procedures. Preoperative patient characteristics are summarized in Table 1. The mean operative time was 102.5 ± 18.2 min, and the average postoperative hospital stay was 5.5 ± 1.6 days. All surgeries were completed without intraoperative complications. Postoperatively, no significant adverse events were observed, and the appearance of the urethral opening was satisfactory. Three months postsurgery, patients exhibited linear urinary stream with no incidence of urinary fistula. The median follow-up duration was 19 months. At 3 months postsurgery, the mean maximum urinary flow rate was 21.2 ± 3.2 ml/s, which decreased slightly to 19.5 ± 4.2 ml/s at 1 year. One patient experienced urine pain with a thin stream and spraying urination after 3 months. Physical examination found a scar at the urethral meatus opening, and the symptoms disappeared after urethral incision. The follow-up survey of sexual life after 1 year showed that seven patients (58.3%) had successful life within 1 year compared with two patients (16.7%) before surgery. There were no cases of erectile dysfunction and poor wound healing. The patients were either very satisfied (75%) or satisfied (25%) with the operation. All patients would recommend urethroplasty to others.

**Table 1:** Characteristics of patients who underwent transurethral reconstruction with annular inlay oral mucosa graft urethroplasty for FN strictures.

Characteristics	Results [mean ± SD or *n* (%)]
Age, years	56.8 ± 6.8
Follow-up duration, months	19.3 + 5.1
Length of urethral stricture, cm	1.6 ± 0.2
Current smoker, *n* (%)	4 (33.3)
Diabetes, *n* (%)	1 (8.3)
Preoperative suprapubic catheter, *n* (%)	1 (8.3)
Stricture etiology, *n* (%)	
Internal trauma	3 (25)
Lichen sclerosus	8 (66.7)
Idiopathic	1 (8.3)

## 4 Discussion

Male urethral stricture remains a prevalent urological challenge with multifactorial etiology and variable therapeutic responses, particularly when involving the anatomically complex urethral meatus and navicularis fossa regions (5). Emerging evidence indicates that LS, characterized by distinct histopathological features including epidermal atrophy, basal layer hydropic degeneration, and chronic lymphocytic infiltration in the superficial dermis, accounts for 38%–69% of anterior urethral strictures according to recent multicenter studies (6). Tan et al. (7) used the next-generation sequencing technology (RNASeq) for miRNA profiling and Ingenuity Pathway Analysis (IPA) for molecular network analysis. It was revealed that differentially expressed miRNAs may play a significant role in the pathogenesis of LS. Our previous study on single-cell RNA sequencing has revealed a unique fibroblastic subset and immune disorder in LS urethral stricture. Specifically, fibroblasts exhibited a distinct subset, designated as Fib7, which is exclusively found in LS urethral stricture disease and demonstrated elevated expression levels of SAA1 and SAA2 (8).

Topical corticosteroids remain the mainstay of conservative management of penile and urethral LS, with current literature supporting the use of other therapies such as tacrolimus and platelet-rich plasma as alternatives or adjuvant treatments when escalation of treatment is necessary (9). For LS affecting genital skin, topical steroids are the primary treatment, but in advanced cases, surgery may be necessary, such as circumcision. When LS causes urethral stricture, urethral dilatation is unlikely to be successful in the long term, and surgery is often required, such as meatoplasty, single- or two-stage urethroplasty, or perineal urethrostomy. Oral mucosal grafting is the preferred grafting method, and the use of genital skin for flaps or grafts is best avoided due to the tendency for recurrence (3, 10).

Urethral meatus and navicularis fossa strictures represent a unique subset of urethral strictures. Despite the relatively short length of this type of urethral stricture, it exhibits a high restenosis rate. Patients have more expectations for the recovery of appearance and sexual function, as well as for minimizing complications (11). Urethrotomy is a classic surgical technique frequently employed in clinical practice. Kulkarni et al. (12) conducted a multicenter study involving 215 male patients with LS leading to anterior urethral stenosis, achieving an overall success rate of 87% (187 out of 215 patients) after a mean follow-up of 56 months. Among these, 15 patients with isolated external urethral meatus stenosis underwent meatotomy, with a success rate of 80% (12 out of 15 patients) at a mean follow-up of 59 months.

Malone et al. (13) described a new technique for meatal stenosis in patients with LS. A total of 19 patients underwent the new operation in a 5-year period. The mean patient age was 42.2 years (range, 6–74), and the mean follow-up was 3 years and 9 months (range, 18 months to 6 years and 7 months). The operation involves dorsal and ventral meatotomies with an inverted V-shaped relieving incision to correct puckering caused by dorsal meatotomy. There were no recurrences or major complications. A total of 13 patients replied to the questionnaires. All patients were pleased or very pleased with the cosmetic result. Eleven patients (out of 13 or 85%) did not spray at all, while the remaining two patients only sprayed occasionally. No patient found it constant or severe.

Despite the high success rate of the initial stage of urethral meatus incision, the procedure has its drawbacks, including abnormal morphology of the external urethral meatus and urinary spraying. Surgical scars and injuries may result in restenosis, and it is not advisable for patients with a long length of scaphoid fossa strictures.

Jordan et al. (14) first used a ventral pedicled flap to repair the stricture of the urethral meatus and navicularis fossa strictures. Literature reported that the success rate of this operation was 83%, and the integrity and beauty of the penile head were preserved. Goel et al. (15) reported urethral meatus plasty using the buccal mucosa transplantation technique and enrolled 10 patients in their study. After penile degloving, the strictured urethra was opened ventrally, and glanular wings were raised. A dorsal urethrotomy incision was performed to create a dorsal bed where an appropriately sized buccal mucosal graft (BMG) was stitched. In the glanular portion, a ventral graft was placed, which is secured ventrally by the glans wings created before. Thus, the glans wings provide a vascular bed to the ventral graft. However, the operation still needs to open the cephalic wing of the penis, which is prone to complications such as bleeding, appearance, and sensory abnormalities of the cephalic penis.

Onol et al. (16) employed circular oral mucosa transplantation to treat 19 patients with distal urethral stricture. Stricture was limited to the glanular urethra (≤2 cm) in all cases, and 16 patients had LS. Stricture urethra was resected 0.5 cm proximal to the healthy urethra, and a rectangular BMG with 4 cm length and 1.5–2.5 cm width (depending on the length of the defect) was rolled on a 24 Fr sound that calibrated the urethra. Proximal and distal edges of the circular BMG were anastomosed circumferentially to the healthy mucosa and meatus, respectively. Foley catheter was removed within 10–14 days. Voiding symptoms, uroflowmetric parameters, and cosmesis were assessed at 1, 3, and 6 months and yearly thereafter. With a median follow-up of 38 months, 16 (84.2%) patients were cured. One patient developed early graft loss, and two patients developed stricture at the proximal anastomotic site. The mean maximum urinary flow rate increased from 7.8 ± 5.4 ml/s preoperatively to 21.8 ± 9.2 ml/s. Although the transurethral approach maintains the integrity of the penile head, it presents challenges in securing the oral mucosa to the normal urethral mucosa with deep and precise sutures. For patients with longer segments of stricture, any urethral sutures exposed to the skin should be excised.

Nikolavsky et al. (17) introduced a fully transurethral technique for transurethral ventral oral mucosa grafting inlay. The procedure involved a transurethral ventral wedge resection of the stenosed segment and construction of an oral mucosa graft shaped like a “teardrop.” Both arms of a double-armed 6-0 PDS suture were passed through the apex of the oral mucosa grafting, through the proximal aspect of the urethrotomy, and externalized through the skin ventrally. The graft was brought into position by pulling on the arms of the externalized suture. Additional double-armed 6-0 PDS sutures were used to quilt the graft into place, securing the distal aspect of the graft to the edge of the meatotomy. The initial description of Tratsurethral ventral buccal mucosa graft inlay urethroplasty included three patients with no stricture recurrences over a mean follow-up of 18 months. A subsequent review of this technique by Daneshvar et al. (18) reviewed 68 patients from 12 institutions, with an average follow-up period of 19 months (ranging from 12 to 49 months). Ninety-five percent of the patients experienced smooth urination without complications such as fistula formation, balanopreputial dehiscence, graft necrosis, or penile curvature. This technique has proven reliable for patients with LS.

Farrell et al. (19) described a technique of transurethral dorsal inlay oral mucosa transplantation urethroplasty. The dorsal urethral opening was incised, and triangular-shaped oral mucosa grafts were secured to the dorsal urethra using 5-0 sutures. The study included 16 patients with a mean age of 63.1 years (range, 43.9–75.6 years) and a mean urethral stricture length of 1.7 cm (range, 1.4–2 cm). The median follow-up duration was 28.8 months (interquartile range, 17.6–38 months). Anatomical success was achieved in 15 out of 16 patients (93.8%), while functional success was observed in all 16 patients (100%). One patient experienced recurrence at 4.2 months postoperatively but did not require additional surgical intervention. Patient satisfaction was very high, with 83.3% reporting they were very satisfied and 16.7% indicating they were satisfied.

In our clinical experience, the previous treatment involving urethroplasty with oral mucosa grafts to replace the urethral meatus and navicularis fossa strictures presented problems. The surgery procedure necessitates an incision on either the dorsal or ventral side of the urethral meatus to ensure patency. The deep space of the urethra is often narrow, making it challenging to maneuver the needle for suturing, complicating the operation, and extending the duration. Additionally, the appearance of the glans after surgery is often unsatisfactory. The risk of restenosis, erectile dysfunction, and paresthesia is significant. We utilized a high-frequency needle-shaped electrode that annular excises the urethral meatus and navicular fossa strictures more easily and precisely along the urethral spongy body. This approach resulted in a larger urethral space. Needle suturing is relatively straightforward and also minimizes trauma ventral side of the penis, thereby reducing nerve damage. This is advantageous for lowering the incidence of postoperative erectile dysfunction and sensory abnormalities. We covered the oral mucosa graft annular inlay to completely replace the urethra. The circumferential oral mucosa graft provides more uniform support, reducing the likelihood of recurrent postoperative stenosis and promoting long-term urethral lumen patency. In this study, we collected clinical data from 12 patients with urethral meatus and navicular fossa stricture who underwent transurethral reconstruction with annular inlay oral mucosa graft urethroplasty in Tangdu Hospital from July 2021 to October 2024. Postsurgical outcomes were favorable. At 3 months postsurgery, the mean maximum urinary flow rate was 21.2 ± 3.2 ml/s, which decreased slightly to 19.5 ± 4.2 ml/s at 1 year. Within 1 year postsurgery, seven patients (58.3%) resumed successful sexual activity compared with two patients (16.7%) preoperatively. No new cases of erectile dysfunction or poor wound healing were observed. The appearance of the glans was satisfactory, and patient satisfaction was high.

The limitations of this study include a relatively small sample size and a median follow-up period of 19 months. To better assess long-term efficacy, extended follow-up will be necessary. Additionally, challenges remain in performing sutures for patients with a longer narrow segment. While research into automated suture devices is ongoing, the mass production and miniaturization of these medical devices are still in developmental stages and may require considerable time to achieve practical application. In the future, advancements in micro-suture instruments and techniques could potentially offer enhanced treatment options for patients with urethral meatus and navicularis fossa strictures.

## 5 Conclusion

The results demonstrated that transurethral reconstruction with annular inlay oral mucosa graft urethroplasty is a viable and effective method for treating urethral meatus and navicularis fossa strictures. The procedure yields satisfactory postoperative outcomes, penile appearance, and sexual function results. All patients expressed satisfaction or high satisfaction with the procedure. Although occasional postoperative scarring of the urethral meatus was observed, no other significant complications were reported. Further studies with larger sample sizes and extended follow-up periods are warranted.

## Data Availability

The original contributions presented in the study are included in the article/Supplementary Material; further inquiries can be directed to the corresponding author.
